# Intraoperative color Doppler during manual vacuum aspiration prevents retained products of conception

**DOI:** 10.1002/ijgo.70810

**Published:** 2026-01-19

**Authors:** Tatsuya Yoshihara, Keito Nakayama, Dai Miyashita, Satoko Sasatsu, Maki Ogi, Yosuke Ono, Osamu Yoshino

**Affiliations:** ^1^ Department of Obstetrics and Gynecology University of Yamanashi Yamanashi Japan

**Keywords:** abortion, color Doppler ultrasonography, retained products of conception, ultrasonography, vacuum curettage

## Abstract

**Objective:**

To investigate whether intraoperative confirmation of the disappearance of uterine cavity blood flow using color Doppler during manual vacuum aspiration (MVA) for missed miscarriage reduces the occurrence of retained products of conception (RPOC).

**Methods:**

We conducted a retrospective cohort study of 202 patients who underwent MVA for missed miscarriage before 12 weeks of gestation at the University of Yamanashi between April 2019 and July 2025. Patients were divided into a flow‐confirmation group, in which intraoperative transvaginal ultrasound with color Doppler was used to confirm the disappearance of blood flow, and a non‐confirmation group. The primary outcome was the occurrence of RPOC diagnosed by postoperative ultrasound. Patient characteristics and surgical variables were compared between groups.

**Results:**

RPOC occurred in 25 of 202 cases (12%). None of the 25 patients in the flow‐confirmation group developed RPOC, whereas 14% of the 177 patients in the non‐confirmation group did (*P* = 0.04). The surgeon's years of experience (2.6 ± 1.6 vs 4.9 ± 4.7 years, *P* = 0.004) and postoperative follow‐up duration (1.9 ± 1.0 vs 3.3 ± 4.3 weeks, *P* = 0.02) were significantly shorter in the flow‐confirmation group, but no other significant differences were found in baseline characteristics or surgical variables.

**Conclusion:**

Intraoperative confirmation of the disappearance of uterine cavity blood flow using color Doppler during MVA is a simple, safe, and effective technique to prevent RPOC. This approach may reduce the need for repeat surgery and postoperative hemorrhage and could be incorporated into standard MVA protocols.

## 1 INTRODUCTION

Surgical evacuation for missed miscarriage is one of the most commonly performed gynecologic procedures worldwide.^1^ Traditionally, dilatation and curettage (D&C) using a sharp curette has been widely performed. However, D&C is known to be associated with serious complications such as uterine perforation, endometrial damage caused by excessive curettage, and the development of Asherman's syndrome.^2^, ^3^, ^4^ The incidence of intrauterine adhesions following D&C has been reported to reach 10%–20%, which may subsequently lead to infertility and menstrual disturbances.^4^

Against this background, manual vacuum aspiration (MVA) has recently attracted attention as a minimally invasive and safe procedure, and it is recommended by international guidelines, including those of the WHO.^5^ Compared with D&C, MVA is associated with a lower risk of intraoperative bleeding and uterine perforation, as well as less endometrial trauma, and has therefore become increasingly accepted as the standard treatment for miscarriage management.^6^, ^7^

On the other hand, retained products of conception (RPOC) are still known to occur in a proportion of cases even after MVA.^8^, ^9^, ^10^ RPOC may lead to postoperative hemorrhage, infection, and the need for repeat surgery, thereby increasing both the physical and psychological burden on patients.^11^, ^12^ In particular, vascularized RPOC identified by color Doppler have been reported to carry a high risk of massive postoperative hemorrhage and the need for emergency hemostasis or repeat surgery, highlighting their significant clinical implications.^11^, ^13^

Attempts have been made to use intraoperative two‐dimensional (2D) transvaginal ultrasound to confirm the absence of residual tissue.^9^, ^10^ However, its sensitivity for detecting small residual tissue remains limited. The addition of color Doppler allows for the visualization of residual blood flow within the uterine cavity, potentially enabling more complete evacuation. However, evidence evaluating the effectiveness of this technique remains scarce.

The aim of this study was to determine whether intraoperative confirmation of the disappearance of uterine cavity blood flow using color Doppler during MVA is effective in preventing the occurrence of RPOC.

## 2 MATERIALS AND METHODS

This retrospective cohort study was conducted at a tertiary care university hospital (University of Yamanashi Hospital, Yamanashi, Japan) between April 2019 and July 2025. All procedures were performed by obstetricians and gynecologists from the same department. A total of 202 patients who underwent MVA for missed miscarriage before 12 weeks of gestation were included, while cases of incomplete miscarriage were excluded. The study was approved by the Institutional Review Board of the University of Yamanashi (approval no.: 2761), and informed consent was obtained from all participants in an opt‐out format.

The protocol for surgical evacuation of missed miscarriage at our institution was as follows. Patients were admitted on the morning of surgery, and cervical dilatation was initiated. After initial dilatation, intravenous anesthesia was administered, and further dilatation was performed using dilators provided in the MVA set, followed by uterine evacuation. Patients were discharged the day after surgery and scheduled for outpatient follow‐up approximately 2 weeks postoperatively. RPOC, when present, was diagnosed during postoperative follow‐up visits.

**FIGURE 1:**
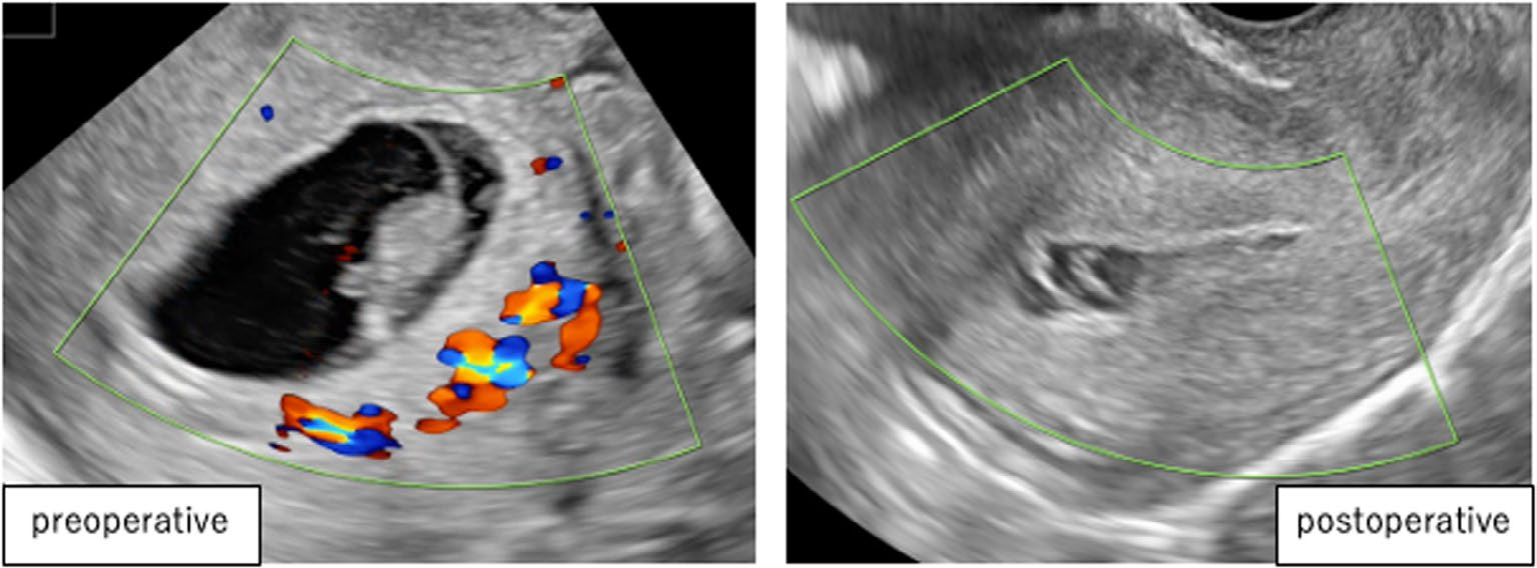
Intraoperative transvaginal ultrasound with color Doppler. Left: Before evacuation, blood flow is detected from the lower part of the image. Right: After evacuation, the intrauterine blood flow has disappeared.

In some cases, intraoperative transvaginal ultrasound (Voluson P8, GE HealthCare, Chicago, Illinois, USA) with color Doppler was performed to confirm the disappearance of blood flow within the uterine cavity (Figure 1). These cases were defined as the “flow‐confirmation group,” whereas those without such confirmation were defined as the “non‐confirmation group.” The use of intraoperative color Doppler was not mandated by an institutional protocol; rather, it was left to the discretion of each operating surgeon. In our department, some surgeons routinely incorporated color Doppler into their MVA procedures, whereas others performed MVA without color Doppler throughout the study period. The decision to use Doppler was based solely on each surgeon's usual practice pattern and was not influenced by resource availability or any specific clinical indications. Thus, allocation to the flow‐confirmation or non‐confirmation group reflected the operating surgeon's usual practice pattern rather than specific clinical indications. The assessment of blood flow disappearance was made in real time by the operating surgeon. The color Doppler settings included a pulse repetition frequency of 0.9–1.8 kHz, a gain adjusted to just below the noise threshold, and wall motion filter of low. Color Doppler assessment was consistently performed immediately after completion of the uterine evacuation to confirm the absence of residual intrauterine blood flow. All intraoperative ultrasound examinations were performed by obstetricians and gynecologists experienced in MVA procedures and transvaginal ultrasonography, who were not blinded to the surgical procedures as they were directly involved in them.

The primary outcome was the occurrence of RPOC after surgery. RPOC was defined as the presence of a hyperechoic area within the uterine cavity detected by transvaginal ultrasound during outpatient follow‐up. The classification of RPOC was based on the Gutenberg classification.^13^ This classification divides RPOC into four types based on grayscale and Doppler ultrasound: type 0 is a hyperechoic avascular mass, type 1 shows mixed echogenicity with minimal vascularization, type 2 is a highly vascularized mass confined to the uterine cavity, and type 3 is a highly vascularized mass with myometrial involvement and possible arteriovenous shunting.^13^ Secondary outcomes included comparisons of patient characteristics and surgical variables.

The patient characteristics evaluated included age, gravidity, parity, body mass index (BMI, calculated as weight in kilograms divided by the square of height in meters), conception by assisted reproductive technology, hormone replacement cycle, history of intrauterine surgery, myoma, adenomyosis, congenital uterine anomaly, cervical conization, and endometriosis. Surgical variables assessed were operative time, intraoperative blood loss, years of obstetric and gynecologic experience of the surgeon, duration of postoperative follow‐up, and the need for repeat surgery. Prior intrauterine surgeries were defined as uterine evacuation or transcervical resection (TCR) of polyps of myomas. Repeat surgery was defined as cases in which complications related to RPOC, such as massive genital bleeding, necessitated repeat uterine evacuation or TCR.

Statistical analyses were performed using JMP software (version 17.2.0). Continuous variables are expressed as mean ± standard deviation (SD). The Chi‐squared, Mann–Whitney *U*, and Fisher exact tests were used as appropriate. A *P* value of less than 0.05 was considered statistically significant.

## 3 RESULTS

The baseline patient characteristics and surgical variables of the 202 cases are summarized in Table 1. During the study period, RPOC occurred in 25 cases (12%). According to the Gutenberg classification, RPOC cases included four type 0, nine type 1, four type 2, and eight type 3 cases.

**TABLE 1:** Baseline characteristics and surgical variables of the study population.

RPOC	25 (12%)	Operative time (min)	10.2 ± 7.1
Age	35.5 ± 5.1	Intraoperative blood loss (mL)	9.8 ± 61.0
Gravidity	2.5 ± 1.4	Years of obstetric and gynecologic experience of the surgeon	4.6 ± 4.5
Parity	0.6 ± 0.8	Postoperative follow‐up duration (weeks)	3.1 ± 4.1
BMI	22.6 ± 3.4	The need for repeat surgery	8 (4.0%)
ART	73 (38%)		
HRC	45 (62%)		
History of intrauterine surgery	77 (38%)		
Myoma	27 (13%)		
Adenomyosis	7 (3.5%)		
Congenital uterine anomaly	4 (2.0%)		
Cervical conization	9 (4.5%)		
Endometriosis	8 (4.0%)		

*Note*: BMI, calculated as weight in kilograms divided by the square of height in meters. All 202 cases included in this study are summarized. Data are presented as mean ± SD or *n* (%). HRC is shown as the proportion within ART pregnancies.

Abbreviations: ART, assisted reproductive technology; BMI, body mass index; HRC, hormone replacement cycle; RPOC, retained products of conception; SD, standard deviation.

There were 25 cases in the flow‐confirmation group and 177 cases in the non‐confirmation group. There were no significant differences between the two groups in gestational age at the time of surgery, history of intrauterine procedures, or the presence of organic uterine or adnexal disorders. Comparisons of RPOC occurrence, patient characteristics, and surgical variables between the two groups are shown in Table 2. The incidence of RPOC was significantly lower in the flow‐confirmation group (flow‐confirmation group; 0% vs non‐confirmation group; 14%, *P* = 0.04). In addition, the years of obstetric and gynecologic experience of the surgeon were significantly shorter (2.6 ± 1.6 years vs 4.9 ± 4.7 years, *P* = 0.004), and the duration of postoperative follow‐up was significantly shorter (1.9 ± 1.0 weeks vs 3.3 ± 4.3 weeks, *P* = 0.02). No significant differences were observed between the two groups in other patient characteristics or surgical variables.

**TABLE 2:** Comparison of patient characteristics and surgical variables between cases with and without RPOC.

	Flow‐confirmation (*n* = 25)	Non‐confirmation (*n* = 177)	*P* value
RPOC	0	25 (14%)	0.04
Age	36.8 ± 4.9	35.4 ± 5.1	0.15
Gravidity	2.4 ± 1.2	2.5 ± 1.4	0.95
Parity	0.8 ± 0.8	0.6 ± 0.8	0.15
BMI	22.5 ± 4.7	22.6 ± 3.2	0.34
ART	10 (40%)	63 (36%)	0.67
HRC	5 (20%)	40 (23%)	0.12
History of intrauterine surgery	5 (20%)	72 (41%)	0.05
Myoma	3 (12%)	24 (14%)	0.83
Adenomyosis	0	7 (4.0%)	0.31
Congenital uterine anomaly	0	4 (2.3%)	0.45
Cervical conization	1 (4.0%)	8 (4.5%)	0.91
Endometriosis	0	8 (4.5%)	0.28
Gestational age	8.0 ± 1.3	8.4 ± 1.2	0.75
Operative time (min)	8.6 ± 4.0	10.4 ± 7.4	0.47
Intraoperative blood loss (mL)	0.6 ± 2.2	11.1 ± 64.9	0.82
Years of obstetric and gynecologic experience of the surgeon	2.6 ± 1.9	4.9 ± 4.7	0.004*
Postoperative follow‐up duration (weeks)	1.9 ± 1.0	3.3 ± 4.3	0.02*
The need for repeat surgery	0	8 (4.5%)	0.25

*Note*: BMI, calculated as weight in kilograms divided by the square of height in meters. Flow confirmation (*n* = 25) and non‐confirmation (*n* = 177) are shown. Data are shown as mean ± SD or *n* (%). Group‐comparison *P* values are from the *χ* ^2^, Fisher exact, or the Mann–Whitney *U* tests, as appropriate (*P < 0.05).

Abbreviations: ART, assisted reproductive technology; BMI, body mass index; HRC, hormone replacement cycle; RPOC; retained products of conception; SD, standard deviation.

## 4 DISCUSSION

The present study demonstrated that intraoperative confirmation of the disappearance of uterine cavity blood flow using color Doppler during MVA may serve as an effective strategy to prevent postoperative RPOC. While no cases of RPOC occurred in the flow‐confirmation group, 14% of cases in the non‐confirmation group developed RPOC, showing a statistically significant difference. These findings suggest a practical intraoperative preventive measure against RPOC, which otherwise occurs in a considerable proportion of cases following MVA.

MVA is primarily performed as a blind procedure, and intraoperative confirmation using conventional 2D transvaginal ultrasound has been considered an effective method to enhance the safety and completeness of the procedure. Indeed, previous studies have reported that ultrasound‐guided MVA may reduce the incidence of RPOC and procedure‐related complications compared with blind techniques.^9^, ^10^ However, 2D ultrasound alone has limitations in detecting small residual tissue or vascularized RPOC. The present study highlights the novelty of incorporating color Doppler, which allows real‐time visualization of intrauterine blood flow and thereby enables the identification of residual tissue that cannot be detected with 2D ultrasound alone.

The findings of this study indicate that evaluating the presence or absence of intrauterine blood flow during surgery is directly linked to the prevention of RPOC which holds significant clinical importance. The use of color Doppler was feasible within a short time frame and did not increase operative time or intraoperative blood loss. Thus, this approach represents a practical method to prevent RPOC without adding invasiveness for patients. Avoiding repeat surgery and postoperative hemorrhage may reduce both the physical and psychological burden on patients and contribute to the efficient use of medical resources. This strategy may be particularly beneficial in patients at high risk of RPOC.

This study had several limitations. First, the flow‐confirmation group included only 25 cases, which limits the statistical robustness of the findings. Second, the allocation into the flow‐confirmation and non‐confirmation groups was not randomized but left to the discretion of the operating surgeon, and therefore potential selection bias cannot be completely excluded. Third, as this was a single‐center retrospective study, the external validity of the results is limited. Furthermore, postoperative reproductive outcomes and perinatal prognoses were not evaluated, which remain important issues for future investigation.

Intraoperative confirmation of the disappearance of uterine cavity blood flow using color Doppler during MVA is a simple and safe technique that may effectively prevent the occurrence of RPOC. The findings of this study position this approach as an advancement of conventional 2D ultrasound‐guided MVA, and its efficacy is expected to be further validated through future multicenter prospective studies. Ultimately, this technique may be incorporated into standard MVA protocols.

## AUTHOR CONTRIBUTIONS

TY: Conceptualization, data curation, formal analysis and writing–original draft. KN: Data collection/management, investigation, writing–review and editing. DM: Formal analysis, methodology, writing–review and editing. SS: Data collection/management, resources. MO: Supervision, methodology, writing–review and editing. YO: Supervision, writing–review and editing. OY: Supervision, project administration, writing–review and editing. All authors approved the final version and agreed to be accountable for all aspects of the work.

## FUNDING INFORMATION

The authors received no specific funding for this work.

## CONFLICT OF INTEREST STATEMENT

The authors declare that there are no conflicts of interest.

## Data Availability

The data that support the findings of this study are available on request from the corresponding author. The data are not publicly available due to their containing information that could compromise the privacy of research participants.
